# Green HPLC method with time programming for the determination of the co-formulated eye drops of tafluprost and timolol in their challengeable ratio

**DOI:** 10.1186/s13065-022-00815-z

**Published:** 2022-04-19

**Authors:** Walaa Nabil Abd-AlGhafar, Fatma Ahmed Aly, Zeinab Awad Sheribah, Samar Saad

**Affiliations:** Faculty of Pharmacy, Pharmaceutical Analytical Chemistry Department, Mansoura University, Mansoura, 35516 Egypt

**Keywords:** Green, Timolol, Tafluprost, HPLC–UV detection-time programming, Eye drops

## Abstract

A new, simple and selective HPLC method was implemented for the simultaneous estimation of tafluprost (TFL) and timolol (TIM) in their new anti-glaucoma combination in the challengeable ratio of 3 and 1000 for TFL and TIM, respectively. Separation was achieved using a BDS Hypersil phenyl column and a mobile phase made up of acetonitrile: 0.015 M phosphate buffer (50:50 v/v, pH 3.5) delivered at 1 mL min^−1^ and the separation was completed in less than 6 min. UV detection was time programmed at 220 nm for the first 4.5 min and later at 254 nm. Mebeverine (MEB) was used as an internal standard (I.S.). The linearity was observed in the ranges of 0.6–45 and 50–2000 µg mL^−1^ with limits of detection (LOD) of 0.18, 16.48 µg mL^−1^ and limits of quantification (LOQ) of 0.55, 49.94 µg mL^−1^ for TFL and TIM, respectively. The method satisfied International Council for Harmonization (ICH) validation guidelines. The study was extended to the estimation of the studied drugs in their co-formulated eye drops as well as in their single dosage forms with acceptable percentage recoveries. Moreover, Green Analytical Procedure Index (GAPI) and analytical Eco-scale were investigated to confirm the greenness of the proposed HPLC method.

**Supplementary Information:**

The online version contains supplementary material available at 10.1186/s13065-022-00815-z.

## Introduction

Glaucoma is a progressive optic neurodegenerative disorder associated with peculiar visual defects and distinct changes in the optic nerve head [1]. It is estimated that 4.5 million people globally are blind due to glaucoma [2]. A popular strategy in the treatment of glaucoma is fixed-dose combination medications, which combine two or more active ingredients in a single dosage form. This strategy is favoured owing to simpler treatment regimens, improved efficacy, patient compliance and superior safety [3]. Prostaglandin analogue and beta-receptor blocker combination therapies are among the most broadly utilized drugs in glaucoma care and are widely used by patients for months or years [4, 5]. Tafluprost is co-formulated with timolol in an ophthalmic formulation. It represents the first preservative-free fixed combination for glaucoma treatment [6, 7]. So, it eliminates the potential side effects associated with the preservatives in ophthalmic formulations and protects the ocular surface.

**Fig. 1:**
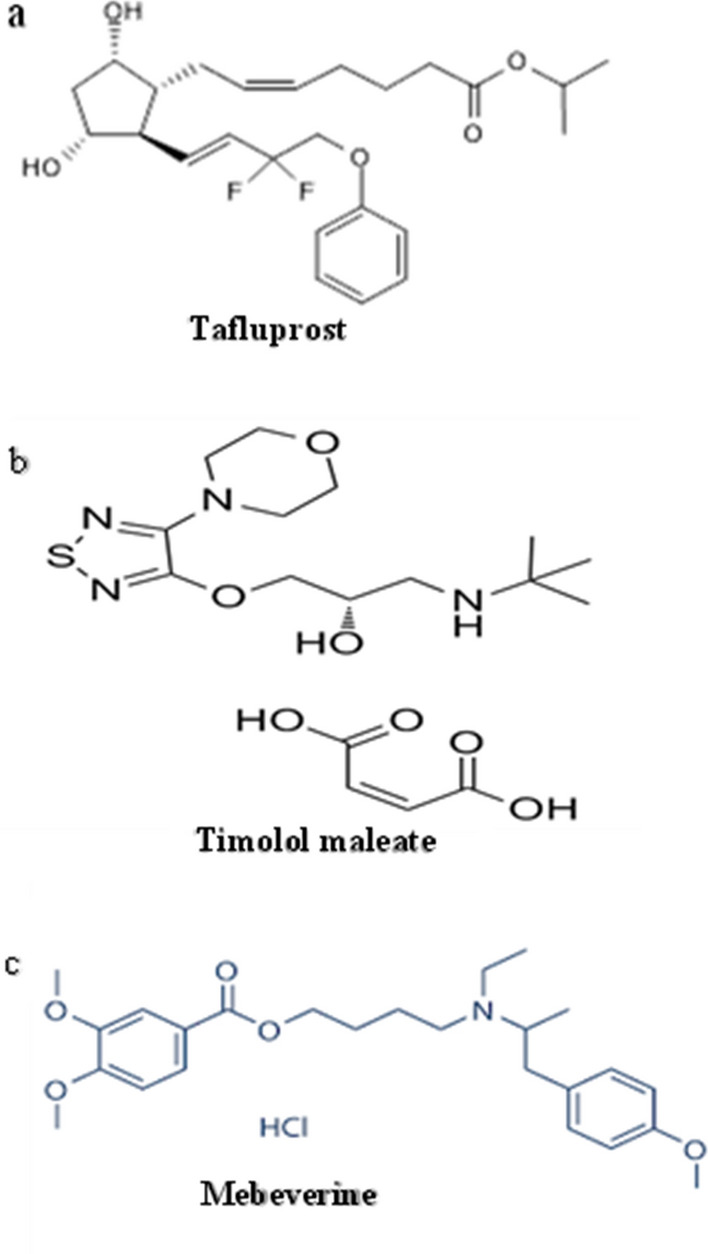
Chemical structure of **a** tafluprost, **b** timolol maleate and **c** mebeverine (I.S.)

Tafluprost is isopropyl-difluoro-4-phenoxybutenyl-dihydroxycyclopentyl-hept-5-enoate (Fig. 1a) [8]. It is a synthetic prostaglandin analogue that lowers the intraocular pressure by increasing the uveoscleral outflow. It is not official in any pharmacopeia. A literature survey reveals HPLC method for disposition and metabolism of TFL following ocular administration to rats [9] and preparative HPLC for TFL purification [10]. Lately, there have been two methods for the estimation of TFL involving: stability- indicating reverse-phase HPLC of bulk drug using a photodiode array [11] and spectrofluorimetry of TFL in its raw material and eye drops [12].

Timolol is 1-(tert-butylamino)-3-[(4-morpholin-4-yl-1,2,5-thiadiazole-3-yl)oxy] propan-2-ol (Fig. 1b) [13]. It is a non-cardioselective β-adrenergic blocker. It works by decreasing the pressure in the eye of patients with glaucoma [14].

BP [13] specified potentiometric titration for the assay of TIM in pure form and direct spectrophotometry for its assay in tablets and eye drops. The USP [15] stated high performance liquid chromatography (HPLC) method for its assay in pure form, eye drops and tablets. Recently, the literature for TIM assay includes: spectrophotometry [16–22], HPLC [23–29], HPTLC [30, 31], chemometry [32, 33], and capillary electrophoresis [34, 35].

TFL and TIM are co-formulated in a challenging ratio (3:1000, w/w), which makes analysis of such a mixture laborious. There is no reported method for the simultaneous determination of TFL and TIM in pharmaceutical preparations. Also, there is a lack of chromatographic methods for the determination of TFL in pharmaceutical samples. This motivates us to establish and validate, for the first time, a simultaneous, new, reliable, easy and fast HPLC method for the estimation of both drugs in synthetic mixtures and eye drops.

HPLC is often preferred in typical laboratories because of its reproducibility, wide availability and suitability. Previous trials in our laboratory were performed to choose a suitable wavelength for detection in HPLC and to resolve the high sensitivity of TIM (high ratio) at the maximum wavelength (220 nm) of TFL (low ratio). The assay of both drugs simultaneously was not possible at the wavelength 220 nm. So, it was promising to switch to time programming technique to allow assay of TFL in the presence of TIM.

In this study, we relied on a simple isocratic mobile phase using phenyl column. The study was performed at room temperature with a total run time of less than 6 min. The suggested HPLC procedure is feasible, green compatible and applied successfully for the first time to analyse both drugs in Taflupro plus® eye drops as well as TFL in its single eye drops with good accuracy and precision. It covers wider linearity ranges for both drugs than the previously documented methods. Also, our method is 350 times more sensitive than the reported HPLC procedure for TFL assay [11], and 5 times more sensitive for TIM than the documented procedure [24] at the same wavelength of UV detection (254 nm).

Since claiming greenness of any analytical approach is insufficient, the suggested procedure was evaluated against two recent greenness tools, GAPI [36] and analytical Eco-scale [37]. Developing and implementing metrics allows the reader to compare the greenness of existing and newly developed studies. The environmental impact of the previously reported methods was not previously evaluated. GAPI [36] is a reliable and semi-quantitative tool, capable of providing a full ecological assessment of the whole analytical technique, from sample collection to final determination. It evaluates 15 factors of each analytical methodology, including sample preparation, storage, collection, reagents, solvents, instrumentation and waste treatment. The visual presentation of GAPI (five pentagrams) makes it easy for the reader to choose the greenest approach for a certain investigation. These pentagrams are colored green, yellow and red to represent low, medium and high impacts on the environment, respectively [38]. All of these advantages of the GAPI tool have led to its widespread usage by analysts in recent years for evaluating method greenness [39–43]. The Eco-scale score depends on penalty points given for each parameter of the analytical approach. Our method achieved a higher score (82) than the reported [11] and official [15] methods (72 and 76, respectively).

## Experimental

### Apparatus

A LC‐20AD prominence liquid chromatograph with an injection valve (20 µL sample loop) and UV–Visible detector model SPD-20A (Shimadzu Corporation, Koyoto, Japan) was utilized for the chromatographic measurements. The apparatus was fitted with a degasser unit (DGU-20A5). Prominence Communications Bus Module (CBM-20A) was utilized to connect the instrument to a PC computer. Data acquisition and analysis of the peaks were achieved on a Shimadzu LC solution software. Consort pH‐meter Model P‐901 (Turnhout, Belgium) was utilized in pH adjustment.

### Materials and reagents

- Timolol maleate and MEB pure samples were attained from Amoun Co. (Cairo, Egypt) and EVA Pharma (Cairo, Egypt), with purities of 99.45% and 100.12%, respectively in accordance with the manufacturer. TFL was purchased from Sigma-Aldrich (Germany) with %purity ≥ 98% pursuant to the certification of the manufacturer method [44].
- The combination of TFL and TIM is available in Taflupro plus® eye drops (batch no. (10)1,019,113) containing TFL 0.0015% and TIM 0.5% (equivalent to 0.68% timolol maleate), product of Orchidia Pharmaceutical Industries (Egypt).
- Saflutan® eye drops, labelled as containing 15 µg mL^−1^ TFL, are a product of Mundipharma Pty Limited, Australia (batch no. 60001-F). Both eye drops should be kept in the refrigerator. Targotimol® eye drops, labelled as containing 5 mg mL^−1^ TIM (equivalent to 6.8 mg mL^−1^ timolol maleate), are a product of Global Advanced Pharmaceuticals, Egypt, batch no. 95406. All the eye drops were purchased from the local pharmacy (Egypt).
- HPLC grade solvents: Acetonitrile and ethanol were attained from Sigma Aldrich Co. (Germany). Methanol was attained from Tedia (USA).
- Phosphoric acid was obtained from Riedel-deHäen (Germany).
- Maleic acid and sodium dihydrogen orthophosphate were attained from ADWIC and El-Nasr Pharmaceutical Chemicals Co. (Egypt), respectively.

### Procedures

#### Standard solutions

Stock solutions were prepared in methanol to contain 100, 5000 and 400 µg mL^−1^ for TFL, TIM and MEB, respectively. The working standard solution of TFL was 15 µg mL^−1^ while TIM and MEB stock solutions were used as working standard solutions. All the solutions were wrapped in aluminum foil and kept either in the refrigerator (TIM and MEB) or the freezer (TFL) [45].

#### Chromatographic conditions

The analytical column was a BDS Hypersil phenyl column. Isocratic binary mobile phase of acetonitrile:0.015 M phosphate buffer (50:50, pH 3.5) delivered at a flow rate 1 ml min^−1^. UV detection was recorded at 220 nm for the first 4.5 min, then 254 nm for the next 6 min at ambient temperature. MEB (100 µg mL^−1^) was used as the internal standard (Fig. 1c).

#### Construction of the calibration graph

To have final concentrations within the range of each drug (0.6–45 µg mL^−1^ for TFL and 50–2000 µg mL^−1^ for TIM), appropriate volumes of working standard solutions were moved separately into a set of 10 mL calibrated flasks. Then, MEB (final concentration 100 µg mL^−1^) was added followed by the addition of the mobile phase to reach the mark. Subsequently, three injection volumes for each concentration were injected into the apparatus loop. The average peak area ratios were plotted against the corresponding concentration of each compound (µg mL^−1^) to attain the calibration curves and the regression equations were derived.

#### Assay of TFL/TIM laboratory-prepared mixtures

Laboratory-prepared mixtures of TFL and TIM in the pharmaceutical ratio of (3:1000) were prepared from the working standard solutions. The procedure under “Construction of the calibration graph” section was carried out. Percentage recoveries were calculated by referring to the previously derived regression equations.

#### Assay of ophthalmic formulations

For Targotimol®: The contents of 30 single bottles of the ophthalmic formulation were mixed and an aliquot of 10 mL (equivalent to 50 mg TIM) was transferred to 25 mL calibrated flask. Then, the mark was reached with methanol. Analysis was done by carrying out the procedure under “Construction of the calibration graph” section.

For Saflutan®: The contents of 30 single bottles were mixed and appropriate volumes (equivalent to 0.015, 0.03 and 0.06 mg TFL) were moved to a series of 10 mL calibrated flasks. MEB (I.S.) was added followed by the addition of the mobile phase to reach the mark and then analysed as mentioned under “Construction of the calibration graph” section.

For Taflupro plus®: The contents of 30 single bottles were mixed and appropriate volumes (equivalent to 0.015, 0.03 and 0.06 mg TFL and 5, 10 and 20 mg TIM, respectively) were moved to a series of 10 mL calibrated flasks. Then, the same procedure under Saflutan® eye drops was carried out.

By utilizing the previously derived regression equations, percentages found were calculated.

## Results and discussion

### Method optimization

**Fig. 2:**
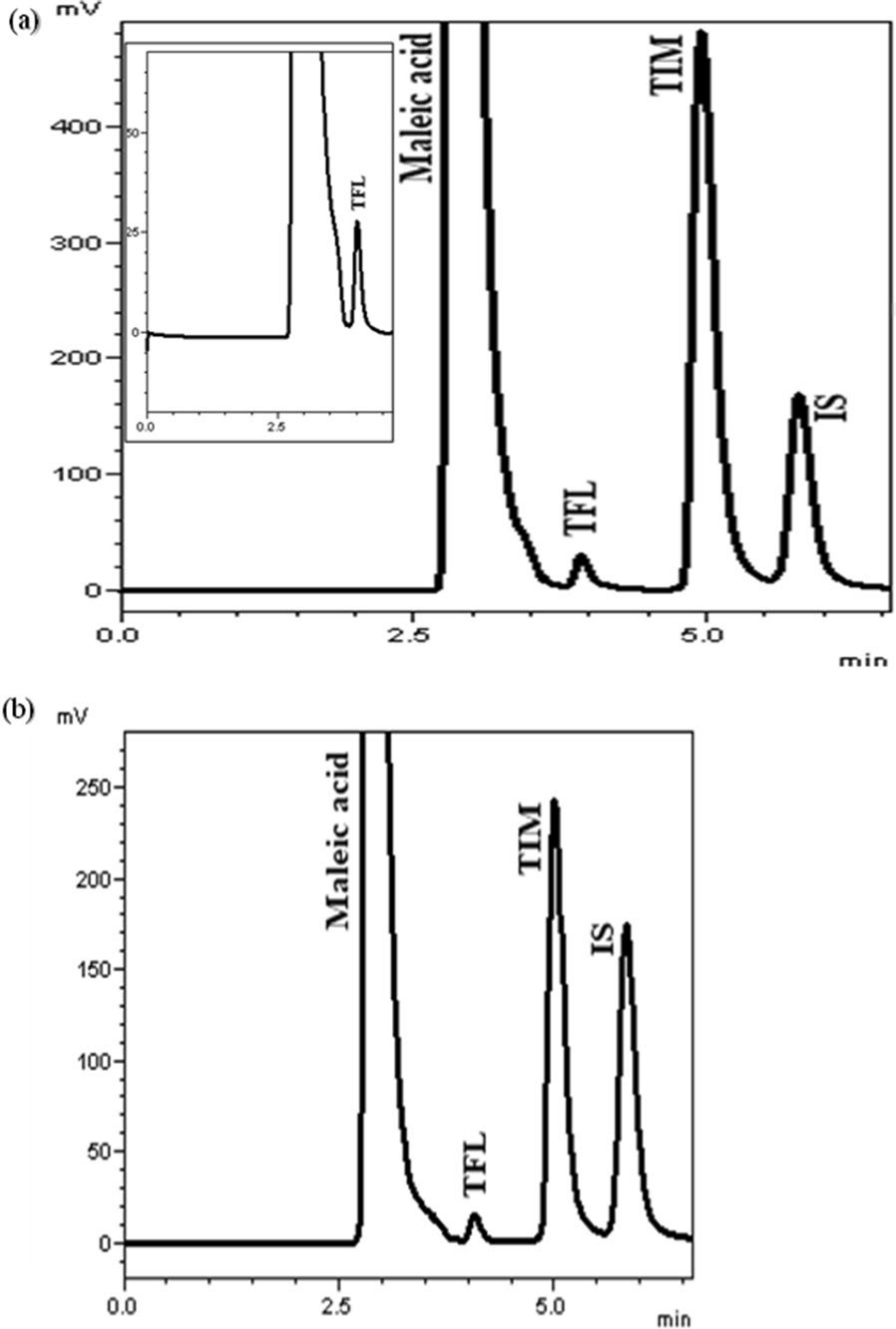
Chromatograms of: **a** laboratory prepared mixture of 6 µg mL^−1^ TFL, 2000 µg mL^−1^ TIM and 100 µg mL^−1^ MEB (IS). **b** Taflupro plus® eye drops 3 µg mL^−1^ TFL and 1000 µg mL^−1^ TIM in the presence of 100 µg mL^−1^ (IS) under the described chromatographic condition

The studied drugs were successfully separated utilizing the developed HPLC procedure coupled with time programming technique. Figure 2 shows the chromatogram for TFL and TIM under the studied HPLC conditions. The performed attempts for method optimization were discussed as follows:

### Choice of column

Primarily, three stationary phases were included in the study:

1. HyperClone™ ODS (C18) column (150 × 4.6 mm i.d., 5 µm).
2. ShimPack (150 × 4.6 mm i.d., 5 µm) CLC-cyanopropyl column.
3. Hypersil BDS phenyl column (250 × 4.6 mm i.d., 5 µm).

The last column was the most appropriate one regarding resolution and efficiency. The first column eluted TIM with the solvent front, while the second column had lower resolution and efficiency than the phenyl column.

It is notable that the phenyl column selectivity varies from the alkyl silica columns. The retention on phenyl column increases as the π-π interactions of the compounds increase in the following order: aliphatic < substituted benzenes < polyaromatic hydrocarbons [46]. Furthermore, the introduction of heteroatoms into the aromatic rings has an obvious enriching effect on their π activity [47]. On this basis, maleic acid, which is an aliphatic moiety, is expected to elute first, followed by TFL (substituted benzene), then TIM (aromatic ring with heteroatoms), and finally MEB (I.S.) (polyaromatic hydrocarbon with heteroatoms).

### Selection of suitable wavelengths

**Fig. 3:**
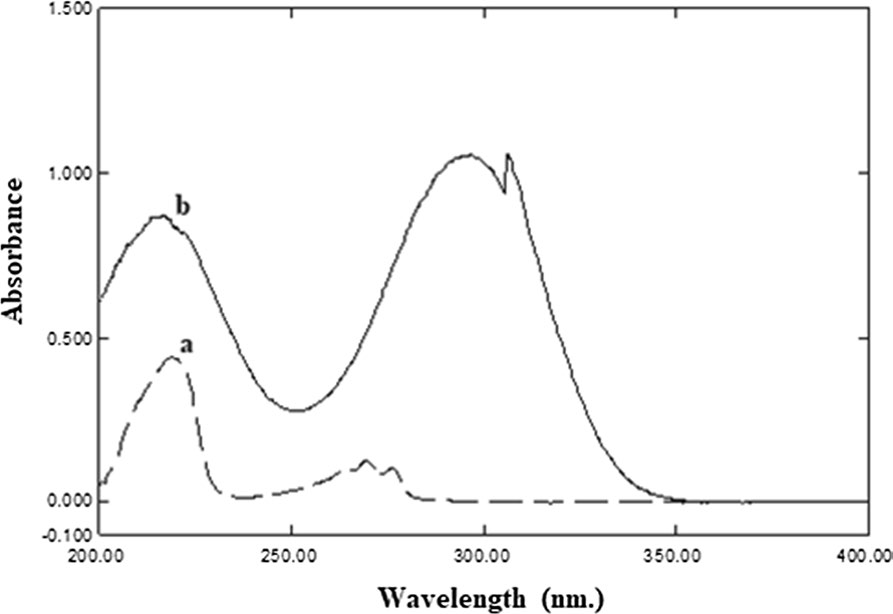
Zero-order absorption spectra of **a** 20 µg mL^−1^ TFL and **b** 50 µg mL^−1^ TIM in methanol

Firstly, the maximum wavelength of TFL (220 nm) was tried as TFL is present in a small concentration in the eye drops (15 µg mL^−1^). It was shown that 220 nm could not be used for the assay of both compounds simultaneously because TIM showed higher absorptivity than TFL at that wavelength (Fig. 3). TIM exhibited maxima in its spectra at 216 and 295 nm. At 254 nm, TIM showed relatively low absorptivity, which allowed the determination of the high ratio of TIM compared to the small ratio of TFL (1000:3). Time programming technique was elected to permit the analysis of TFL with good sensitivity concurrently with reasonable TIM sensitivity. The wavelength of 220 nm was set for detection of TFL, whilst 254 nm was set for detection of TIM. Maleic acid (the salt moiety of TIM) was eluted after 2.8 min and was detected at 220 nm. To confirm the identity of maleic acid, pure maleic acid solution was injected and it appeared at the same retention time.

### Mobile phase composition

Several trials in the mobile phase were studied to accomplish better results in the chromatographic system. These trials are:

### pH

The effect of pH was studied over the range of 3.0–6.0. It was noticed that pH adjustment insignificantly affects the retention time of both compounds. This action was presumably attributed to the high pK_a_ of them (pK_a_ = 9.21 [48] and 14.51 [49] for TIM and TFL, respectively). So, both compounds are in the cationic forms over the studied pH range. pH 3.5 was chosen due to the best resolution and the highest theoretical plates (Additional file 1: Table S1).

### Type and ratio of the organic solvent

Acetonitrile, methanol and ethanol were tried. Methanol led to peak broadening, decreased efficiency, and increased retention time of both compounds (t_R_ = 6.14 and 12.94 min for TFL and TIM, respectively). Ethanol led to overlapped TFL and TIM peaks. Acetonitrile was premium as its use in 50% gave well resolved peaks in a short analysis time (less than 6 min). Increased ratios of more than 50% resulted in inadequate separation of TFL from maleic acid, while decreasing ratios of less than 50% led to inadequate separation of TFL from TIM (Additional file 1: Table S1).

### Ionic strength of buffer

Different ionic strengths of phosphate buffer were tried. Decreasing ionic strength of the buffer (less than 0.015 M) led to a long run time, while increasing ionic strength (more than 0.02 M) led to overlapped TFL and TIM peaks. Eventually, 0.015 M was chosen for the study as it combines good resolution, theoretical plates and analysis time (Additional file 1: Table S1). It was noticed that using water alone instead of phosphate buffer led to a significant decrease in the sensitivity and efficiency of both compounds.

### Flow rate

Finally, the impact of flow rate was tested in the range of 0.6–1.2 mL min^−1^. A flow rate of 1 mL min^−1^ was chosen in the study, as it was associated with the highest theoretical plates within a reasonable retention time. A flow rate of less than 1 mL min^−1^ led to a long retention time, whilst a flow rate of 1.2 ml min^−1^ resulted in lower efficiency and resolution between TFL and maleic acid (Additional file 1: Table S1).

### Internal standard selection

Using an internal standard is important in ensuring the accuracy and precision of the quantitative analysis [50]. Several compounds that could elute under the same chromatographic conditions were tried to choose the best one. These compounds are: valsartan, labetalol, betaxolol, diazepam and MEB. Mebeverine was chosen as the best internal standard as it provided good resolution (R_s_ between TIM and MEB = 2.67). Betaxolol overlapped with TIM, and the others had poor resolution.

### Method validation

#### Linearity and range of concentration

Regression plot showed a linear dependence of the attained peak area ratios on the drug concentrations (µg mL^−1^). The graph was linear over the ranges stated in Table 1. The linear equations were as follows:

$$\text{P }=-0.0\text{495 }+\text{ }0.00\text{18 C }\left( \text{r }=\text{ }0.\text{9999} \right)\text{ for TIM}$$

$$\text{P }=\text{ }0.000\text{4 }+\text{ }0.0\text{159 C }\left( \text{r }=\text{ }0.\text{9999} \right)\text{ for TFL}$$

where﻿ P is the attained peak area ratio, C is the concentration of the drug (µg mL^−1^) and r is the correlation coefficient. Regression data mentioned in Table 1, illustrates the linearity of the studied procedure.

**Table 1:** Analytical performance data for the adopted method for the estimation of TIM and TFL

Parameter	Proposed method	
	TFL	TIM
Detection wavelength (nm)	220	254
Linearity range (µg mL^−1^)	0.6–45	50–2000
Intercept (a)	0.0004	− 0.0495
Slope (b)	0.0159	0.0018
n	9	8
Correlation coefficient (r)	0.9999	0.9999
Standard deviation of residuals (S_y/x_)	2.0 × 10^–3^	15.6 × 10^–3^
Standard deviation of intercept (S_a_)	9.0 × 10^–4^	9.0 × 10^–3^
Standard deviation of slope (S_b_)	4.0 × 10^–5^	1.0 × 10^–5^
% Relative standard deviation (% RSD)	0.92	1.11
% Error	0.306	0.394
Limit of detection (LOD) (µg mL^−1^)	0.18	16.48
Limit of quantitation (LOQ) (µg mL^−1^)	0.55	49.94

#### Limit of detection (LOD) and limit of quantification (LOQ)

The LOD and LOQ values were calculated using calibration standards as reported by ICH guidelines [51]. These values were calculated as LOD = 3.3 σ/S and LOQ = 10 σ/S, where S is the slope of the calibration graph and σ is the standard deviation of *y*-intercept of regression equation (Table 1).

#### Accuracy

To check the accuracy, average recoveries were calculated after analyzing each drug in raw materials as well as in synthetic mixtures. The assay results of TFL and TIM raw materials were 100.03 ± 0.92 and 100.07 ± 1.11, respectively. Table 2 demonstrates the assay data in synthetic mixtures. Acceptable recoveries with low standard deviations indicate the accuracy of the method [49]. These recovery data were compared to those obtained using the manufacturer [44] and official [15] methods. The manufacturer method for TFL assay relies on gradient HPLC utilizing a mixture of (A) 0.1% trifluoroacetic acid in water and (B) 0.1% trifluoroacetic acid in acetonitrile as mobile phase and C18 column with UV detection at 220 nm [44]. The official procedure for TIM assay in raw material also relies on gradient HPLC, utilizing a mixture of (A) 0.05% trifluoroacetic acid in water and (B) 0.05% trifluoroacetic acid in acetonitrile as the mobile phase. While the official procedure for its assay in eye drops utilizes methanol: phosphate buffer pH 2.8 (35: 65 v/v) at 40 ^◦^C. The column was C18 and UV detection at 295 nm in both raw material and eye drops [15]. The difference in the mean percent found (*t*‐test) or in the variance (F-test) was not statistically significant between the studied method and the manufacturer or official ones [52] (Table 2).

**Table 2:** Assay results for the estimation of the studied drugs in different laboratory prepared mixtures in their pharmaceutical ratios

Parameter	Proposed method						Manufacturer and reference methods [15, 44]	
	Amount taken (µg mL^−1^)		Amount found (µg mL^−1^)		% found		% found	
	TFL	TIM	TFL	TIM	TFL	TIM	TFL	TIM
	1.5	500.0	1.471	490.278	98.07	98.06	98.71	98.62
	3.0	1000.0	2.987	981.444	99.58	98.19	101.11	101.65
	6.0	2000.0	5.887	1995.944	98.11	99.79	99.80	99.41
X̅ ± S.D					98.59 ± 0.86	98.68 ± 0.96	99.87 ± 1.20	99.89 ± 1.57
*t*					1.50 (2.78)	1.14 (2.78)		
F					1.95 (19.00)	2.67 (19.00)		

Each result is the average of three replicate estimations

The theoretical *t* and F values (P = 0.05) are between parentheses [52]

#### Precision

Three concentrations of both TIM and TFL were analyzed three times each on the same day (intra-day) and the precision was calculated as %RSD for the studied method. A comparable proceeding was compassed to check the inter-day precision but on three separate days. The obtained %RSD values were less than 2% indicating the high repeatability and inter-day precision.

#### Specificity

The specificity of the methodology was corroborated by the estimation of both TIM and TFL in the commercial eye drops. No interference was noticed from the eye drops excipients (glycerol, sodium dihydrogen phosphate, disodium edetate, tween 80) [53] (Table 3).

**Table 3:** Assay results for the estimation of the studied drugs in their co-formulated and single eye drops

Ophthalmic formulation	Proposed method				Manufacturer and reference methods [15, 44]	
	Amount taken (µg mL^−1^)		% found		% found	
Saflutan® (contains TFL 15 µg mL^−1^)	1.5		98.07		101.71	
	3.0		99.58		98.13	
	6.0		98.11		100.94	
X̅ ± S.D			98.59 ± 0.86		100.26 ± 1.88	
*t*			1.40 (2.78)			
F			4.78 (19.00)			
Targotimol® (contains TIM 5 mg mL^−1^)	80.0		101.94		98.90	
	250.0		99.89		101.36	
	1000.0		98.03		99.51	
X̅ ± S.D			99.95 ± 1.96		99.92 ± 1.28	
*t*			0.02 (2.78)			
F			2.34 (19.00)			

Taflupro plus® (contains TFL 15 µg mL^−1^ and TIM 5 mg mL^−1^)	TFL	TIM	TFL	TIM	TFL	TIM
	1.5	500	98.11	98.17	98.02	100.81
	3.0	1000	98.11	98.08	100.98	98.54
	6.0	2000	99.16	99.43	99.81	100.92
X̅ ± S.D			98.46 ± 0.61	98.56 ± 0.75	99.57 ± 1.45	100.09 ± 1.34
*t*			1.22 (2.78)	1.73 (2.78)		
F			5.65 (19.00)	3.19 (19.00)		

Each result is the average of three replicate estimations

The theoretical t and F values (P = 0.05) are between parentheses [52]

#### Robustness

The robustness of the method was studied to prove that it is reliable under slightly varied conditions. These variations were pH (3.5 ± 0.1), acetonitrile ratio (50 ± 1%) and ionic strength of the phosphate buffer (0.015 ± 0.005). The peak area ratios of TIM and TFL were not significantly influenced by these slightly changed conditions (Additional file 1: Table S2).

#### System suitability

HPLC parameters (NTP, R_s_ and T) were calculated to check system suitability. The values were within acceptable ranges regarding USP [15] and ICH guidelines [51] which prove the method’s system suitability. Theoretical plates were 4681 and 3108, and tailing factors were 1.502 and 1.433 for TFL and TIM, respectively. The resolution between both drugs was 3.034.

### Method application

#### Laboratory prepared mixtures and eye drops assay

Good %recoveries with small values of S.D. (Tables 2 and 3) corroborate the appropriateness of the suggested methodology for the quantification of TIM and TFL in laboratory prepared mixtures, co-formulated eye drops and in their single eye drops. The results obtained concurred with those of the manufacturer [44] and the official [15] methods, as verified by the *t* and F values [52] (Fig. 2b).

#### Comparison to the published and reference procedures

It is the first time to establish an analytical procedure for the assay of TIM and TFL simultaneously. It offers advantages over the reported HPLC procedure [11] for the assay of TFL. The studied HPLC procedure is more sensitive with LOD and LOQ (0.18 and 0.55 µg mL^−1^) compared to (57.5 and 210.5 µg mL^−1^) in the reported one. Our HPLC method is found to be superior as it is much quicker (TFL was eluted in 4 min compared to 22 min in the reported one [11]) and it uses much lower solvent quantities, which leads to reducing cost and improving the safety of the environment and the analyst. Besides, the studied isocratic HPLC eliminates the need for column temperature control, providing the benefit of energy savings, compared to performing at 50 °C in gradient elution in the reported one [11]. The assay of the commercial eye drops that contain a very low TFL concentration was not studied in the reported HPLC [11], while they were successfully estimated in our procedure with % recovery of 98.59 ± 0.86.

Also, the proposed procedure is simpler than the USP official procedure for TIM assay in raw material [15]. The latter depends on gradient elution, which requires a more expensive special pump. The diluent in that method [15] is methanol: water (60: 40 v/v) and the column temperature is maintained at 40 °C.

#### Greenness evaluation

**Fig. 4:**
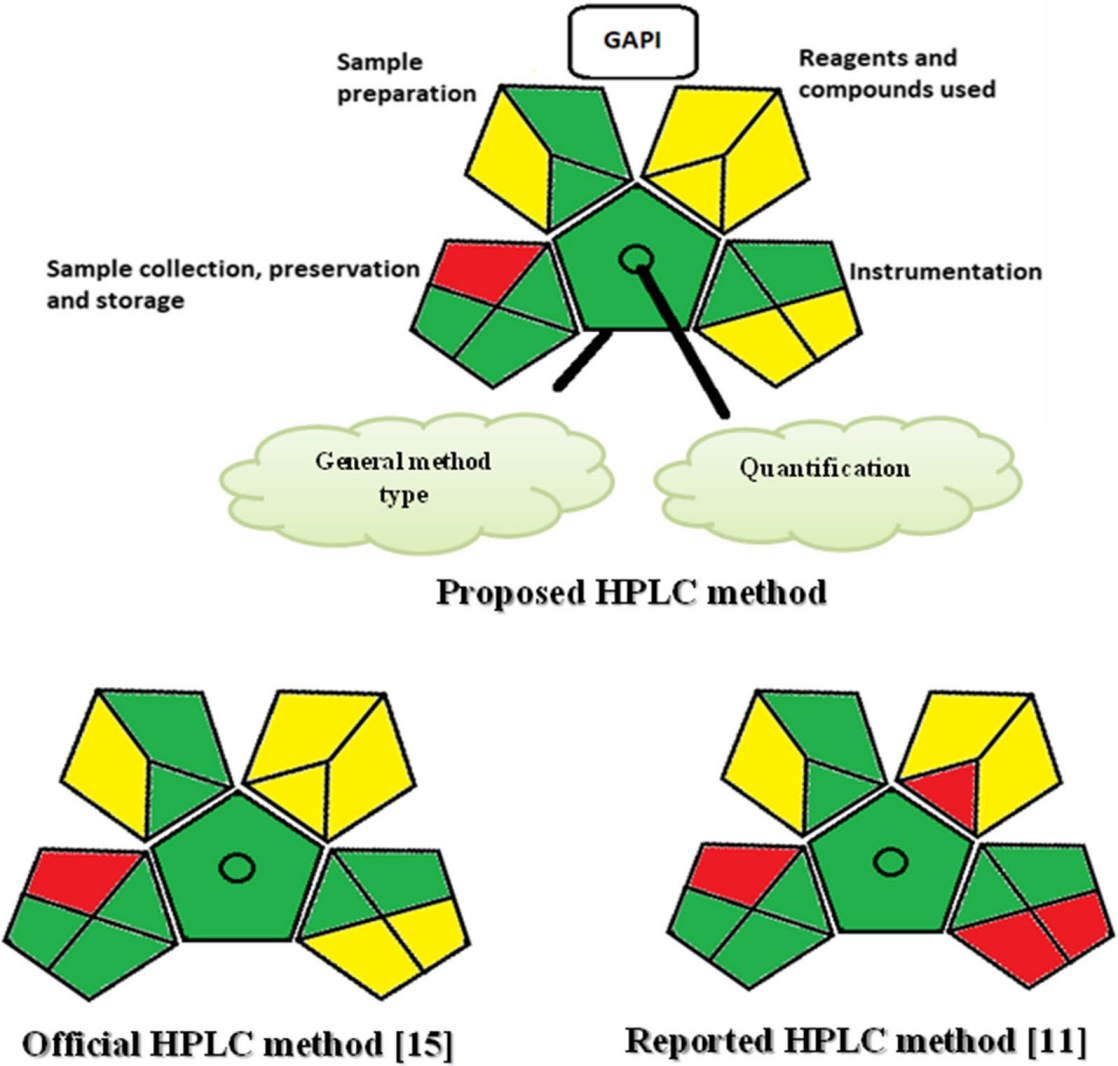
The green evaluation profile for the proposed HPLC method in comparison with the reported (for TFL assay) [11] and official (for TIM assay) [15] HPLC methods, using the GAPI tool [36]

The evaluation of the ecological impact for newly developed analytical methodology has become an important feature during method development to provide a brief and objective assessment for future comparisons between published methods. Two approaches were used to investigate the greenness of the suggested method (GAPI and analytical Eco-scale). The suggested approach has a low number of steps and does not require any specific preparation conditions, resulting in a reduction in time and energy consumption. GAPI assessment tool of the suggested, reported [11] and reference [15] HPLC methods is presented in Fig. 4.

Additionally, analytical Eco-scale [37] was introduced so as to assess the greenness of the suggested method with the reported [11] and reference [15] HPLC ones as shown in Table 4. It evaluates the greenness depending on penalty points. The ideal score of the method is 100. The Eco-scale total score is measured by subtracting all of the penalty points of the method’s parameters from the ideal score. The closer the score to 100 is, the greener the procedure becomes. The score of our method was 82, while in the reported [11] and reference [15] ones were 72 and 76, respectively.

**Table 4:** Analytical Eco-scale penalty points [37] of the proposed method against the reported and USP HPLC methods

Proposed HPLC method		Reported HPLC method [11]		Reference HPLC method [15]	
Reagents	Penalty points	Reagents	Penalty points	Reagents	Penalty points
Acetonitrile	8	Acetonitrile	8	Acetonitrile	8
0.015 M Phosphate buffer	0	Methanol	6	Methanol	6
Internal standard	4	Water	0	Water	0
		0.1% orthophosphoric acid	4	0.05% trifluoroacetic acid	4
	∑12		∑18		∑18

Instruments	Penalty points	Instruments	Penalty points	Instruments	Penalty points
Transport	1	Transport	1	Transport	1
HPLC–UV detection	2	HPLC–UV detection	2	HPLC–UV detection	2
Occupational hazard	0	Occupational hazard	0	Occupational hazard	0
		Heater (50 ^◦^C)	2	Waste	3
Waste	3	Waste	5		
	∑6		∑10		∑6
Total penalty points	18	Total penalty points	28	Total penalty points	24
Score	82	Score	72	Score	76

It is concluded from the preceding approaches that the investigated HPLC procedure is an excellent green one.

## Conclusion

Ocular medications for glaucoma should be administered in the correct dose to ensure optimal efficacy and avoid patient side effects. Timolol is co-formulated with TFL in eye drops in a challenging ratio (1000:3). So, there is a need to implement a simple, accurate, precise and sensitive HPLC procedure to overcome the problem raised by that ratio and allow analyzing both medications simultaneously. The suggested approach has various advantages being the first one for concurrent separation, reproducible, wide linearity ranges and has short retention time (less than 6 min). The HPLC technique was assessed as a whole approach and regarded inexpensive thanks to the comparatively low cost of the used mobile phase and the isocratic elution mode. HPLC–UV apparatus is relatively available in many laboratories. The suggested method was successfully applied in real life situations by the estimation of both drugs in Taflupro plus® eye drops as well as in their single eye drops with acceptable percentage recoveries. It was compared to the reported HPLC procedure (for TFL assay) and USP official procedure (for TIM assay) for greenness assessment using two different tools and it was obvious that our method was greener. This encourages our suggested approach to be employed as an efficient, easy and eco-friendly analytical tool for routine high-throughput analysis required in research centers and quality control laboratories to ensure that precise doses of both drugs are administered. In addition to promoting the proposed approach to be carried out in pharmacokinetic studies.

## Supplementary Information

**Additional file 1:** **Table S1.** Optimization of the chromatographic conditions for the estimation of TIM and TFL using the studied HPLC method. **Table S2.** Robustness of the studied HPLC method for the determination TIM (1000 µg mL-1) and TFL (3 µg mL-1).

## Data Availability

All the data generated or analysed during this study are included in this article and its Additional file 1.
