# Supplementary material for: Green HPLC method with time programming for the determination of the co-formulated eye drops of tafluprost and timolol in their challengeable ratio
Source: BMC Chem. 2022 Apr 19;16(1):28. doi: 10.1186/s13065-022-00815-z (PMC9019973; doi:10.1186/s13065-022-00815-z)
Supplement: Supplementary file 1 — Additional file 1: Table S1. Optimization of the chromatographic conditions for the estimation of TIM and TFL using the studied HPLC method. Table S2. Robustness of the studied HPLC method for the determination TIM (1000 µg mL-1) and TFL (3 µg mL-1). [file 13065_2022_815_MOESM1_ESM.docx]

**Table S1** Optimization of the chromatographic conditions for the estimation of TIM and TFL using the studied HPLC method.

| Parameter | Number of theoretical plates (NTP) | | Retention time (t_R_) (min) | | Resolution (R_s_) | | Tailing factor (T) | |
| --- | --- | --- | --- | --- | --- | --- | --- | --- |
|  | TIM | TFL | TIM | TFL | Maliec acid/TFL | TFL/TIM | TIM | TFL |
| pH of the mobile phase 3.0  3.5  4.0  5.5  6.0 | 2604  422s8  4070  3901  3891 | 3017  3018  1521  1553  1495 | 6.54  4.71  4.60  4.56  4.63 | 5.04  4.01  3.78  3.63  3.73 | 7.613  4.508  3.065  2.715  2.975 | 3.741  2.405  2.447  2.801  2.660 | 1.562  1.502  1.557  1.575  1.587 | 1.740  1.455  1.166  1.455  1.200 |
| Acetonitrile:phosphate buffer ratio (v/v)  40:60  50:50  60:40  70:30 | 3284  4681  4900  5707 | 4885  3108  1728  2844 | 6.05  5.10  4.89  4.73 | 5.33  4.10  3.55  3.31 | 10.108  5.405  2.596  2.062 | 1.974  3.034  4.296  5.672 | 1.524  1.511  1.457  1.472 | 1.564  1.433  1.187  1.077 |
| Ionic strength of phosphate buffer  (M) 0.005  0.010  0.015  0.020  0.03  0.04 | 5461  5021  4681  4228 | 2265  2661  3108  3018 | 7.72  5.82  5.10  4.71 | 3.84  3.94  4.10  4.01 | 5.039  4.841  5.405  4.508 | 10.481  5.920  3.034  2.405 | 1.544  1.493  1.511  1.533 | 1.378  1.472  1.433  1.521 |
|  | TIM and TFL peaks are overlaped | | | | | | | |
| Effect of flow rate (mL min^-1^) 0.6  0.8  1.0  1.2 | 5887  5493  4681  4679 | 3511  3222  3233  3001 | 8.25  6.22  5.10  4.22 | 6.45  4.89  4.10  3.32 | 5.036  4.870  5.405  4.512 | 4.172  3.932  3.034  3.669 | 1.595  1.586  1.511  1.512 | 1.432  1.412  1.433  1.434 |

Number of theoretical plates (NTP) = $5.54 \left( \frac{t_{R}}{W_{h/2}} \right)^{2}$[15], R_s_ = $\frac{2\Delta t_{R}}{W_{1}+ W_{2}}$ [15]

Tailing factor (T) = ${W_{0.05}}/{2f}$ [15]

**Table S2** Robustness of the studied HPLC method for the determination TIM (1000 µg mL^-1^) and TFL (3 µg mL^-1^).

| Parameter | Amount found  (µg mL^-1^) | | %found | | Retention time (t_R_)(min) | | Tailing factor (T) | |
| --- | --- | --- | --- | --- | --- | --- | --- | --- |
|  | TFL | TIM | TFL | TIM | TFL | TIM | TFL | TIM |
| Acetonitrile ratio%  49  50  51 | 3.057  2.981  2.956 | 994.167  1009.426  983.056 | 101.89  99.37  98.53 | 99.42  100.94  98.31 | 4.16  4.10  4.06 | 5.06  5.10  5.06 | 1.29  1.17  1.10 | 1.79  1.50  1.77 |
| X̅ ± S.D. | 99.93 ± 1.75 | | | 99.56 ± 1.32 | | | | |
| %RSD | 1.75 | | | 1.33 | | | | |
| pH of the mobile phase  3.4  3.5  3.6 | 3.057  2.981  3.025 | 980.278  1009.426  983.056 | 101.89  99.37  100.84 | 98.03  100.94  98.31 | 4.16  4.10  4.14 | 5.04  5.10  5.07 | 1.32  1.17  1.30 | 1.79  1.52  1.70 |
| X̅ ± S.D. | 100.70 ± 1.27 | | | 99.09 ± 1.61 | | | | |
| %RSD | 1.26 | | | 1.62 | | | | |
| Ionic strength of phosphate buffer (M)  0.01  0.015  0.02 | 3.019  3.050  3.044 | 994.167  1001.389  1010.833 | 100.63  101.68  101.47 | 99.42  100.14  101.08 | 3.94  4.19  4.01 | 5.50  5.10  4.91 | 1.47  1.43  1.49 | 1.49  1.51  1.54 |
| X̅ ± S.D. | 101.26 ± 0.56 | | | 100.21 ± 0.83 | | | | |
| %RSD | 0.55 | | | 0.83 | | | | |

Each result is the average of three replicate estimations.
